# The interplay between obesity and cancer: a fly view

**DOI:** 10.1242/dmm.025320

**Published:** 2016-09-01

**Authors:** Susumu Hirabayashi

**Affiliations:** 1 Metabolism and Cell Growth Group, MRC Clinical Sciences Centre (CSC), Du Cane Road, London W12 0NN, UK; 2 Institute of Clinical Sciences (ICS), Faculty of Medicine, Imperial College London, Du Cane Road, London W12 0NN, UK

**Keywords:** Cancer, *Drosophila*, Obesity

## Abstract

Accumulating epidemiological evidence indicates a strong clinical association between obesity and an increased risk of cancer. The global pandemic of obesity indicates a public health trend towards a substantial increase in cancer incidence and mortality. However, the mechanisms that link obesity to cancer remain incompletely understood. The fruit fly *Drosophila melanogaster* has been increasingly used to model an expanding spectrum of human diseases. Fly models provide a genetically simpler system that is ideal for use as a first step towards dissecting disease interactions. Recently, the combining of fly models of diet-induced obesity with models of cancer has provided a novel model system in which to study the biological mechanisms that underlie the connections between obesity and cancer. In this Review, I summarize recent advances, made using *Drosophila*, in our understanding of the interplay between diet, obesity, insulin resistance and cancer. I also discuss how the biological mechanisms and therapeutic targets that have been identified in fly studies could be utilized to develop preventative interventions and treatment strategies for obesity-associated cancers.

## Abstract (precis)

**Summary:** This Review highlights a *Drosophila* model of diet-induced obesity and cancer, and how these two models are combined to study the interplay between obesity and cancer.

## Introduction

The prevalence of obesity is rapidly growing worldwide. According to the World Health Organization, the global incidence of obesity has more than doubled since 1980, reaching 600 million in 2014 (http://www.who.int/mediacentre/factsheets/fs311/en/). Obesity affects whole-body metabolic homeostasis, leading to a number of co-morbidities, including diabetes and cardiovascular diseases (Guh et al., 2009). Furthermore, epidemiological studies have provided strong evidence of an association between obesity and the increased risk of various cancers (Calle et al., 2003; Renehan et al., 2008). A projection study estimates that the current increase in obesity rates could lead to a nearly 15-fold increase in cancer incidence in the United States by 2030 (http://www.cancer.gov/about-cancer/causes-prevention/risk/obesity/obesity-fact-sheet).

Despite the huge impact of the obesity-cancer link on public health and its associated economic burden, the biological links between obesity and cancer remain understudied. This is partly because of the need for a whole-animal approach in studying the link between obesity and cancer; systemic responses induced by obesity could affect tumor growth and progression at multiple levels. Although mouse models are an invaluable whole-animal system for studying cancer in the context of whole-body metabolism, mechanistic genetic analyses are complicated by gene redundancy. In addition, the time and expense of creating mouse models with multiple genetic manipulations constitute an impediment when studying complex disease interactions. Simpler genetic models would be better suited for an initial dissection of the complex interplay between obesity and cancer.

Over the past decade, the fly has emerged as an increasingly popular model system for studying human disease, including neurodegenerative disorders (Bilen and Bonini, 2005; Lu and Vogel, 2009), metabolic diseases (Alfa and Kim, 2016; Owusu-Ansah and Perrimon, 2014) and cancer (Gonzalez, 2013). Up to 75% of human disease-related genes have homologs in *Drosophila* (Reiter et al., 2001). The signaling pathways that regulate growth, differentiation and metabolism are highly conserved between mammals and *Drosophila*. The fly genome contains roughly the same number of genes as the human genome but with less redundancy, which can simplify genetic analyses of disease mechanisms in a whole-animal context. Powerful genetic manipulations that are routinely used in flies, including the GAL4-UAS system (Brand and Perrimon, 1993) and the FRT-FLP recombination techniques (Xu and Rubin, 1993) (see Box 1), are a key strength of *Drosophila* as a model organism. The ability to perform genetic and chemical screens in a whole-animal setting provides a powerful systemic approach. The ease with which such screens can be performed in *Drosophila* makes it a useful model for studying disease mechanisms. Insights from fly studies can then be used to ask more focused questions in mammalian models.

**Box 1. Glossary**

**FLP-FRT recombination system:** This genetic technique is used to generate discrete patches of homozygous mutant clones in an otherwise wild-type tissue. It employs the yeast site-specific recombinase flippase (FLP), which mediates the mitotic recombination of the chromosome arm distal to the FLP recognition target (FRT) sequence.

**GAL4-UAS system:** An approach used in *Drosophila* to activate genes in specific tissues. It utilizes the yeast transcriptional activator GAL4 and its binding site, the upstream activating sequence (UAS), which is fused to a gene of interest. To activate the fused UAS gene, flies are crossed to other flies that express GAL4 in specific tissues.

**MARCM:** The mosaic analysis with a repressible cell marker (MARCM) technique generates marked homozygous mutant clones induced by the FLP-FRT-mediated recombination system and simultaneously expresses the genes of interest by using the GAL4-UAS system in an otherwise wild-type tissue.

Several hypotheses concerning the underlying mechanisms that link obesity and cancer have been proposed, including hormonal effects (involving insulin), metabolic effects (involving glucose) and inflammation, as well as the role of gut microbial metabolites. A key area of focus for research into the obesity-cancer connection is the role of the increased insulin levels that are associated with obesity (Cohen and LeRoith, 2012). However, how tumors utilize the increased levels of circulating insulin in an insulin-resistant organism remains an unsolved question. In this Review, I summarize recent developments with respect to *Drosophila* diet-induced obesity models and genetically engineered cancer models, and how the combination of these models has been used to understand the interplay between obesity and cancer. I also highlight some of the outstanding questions and future directions that have emerged from these fly studies.

## Linking obesity and cancer

Epidemiological studies have indicated that obesity is not only a risk factor for diabetes and heart disease, but that it also increases the risk of several types of cancer (Calle and Kaaks, 2004; Renehan et al., 2008). In this section, I summarize epidemiological studies that connect obesity to the risk of cancer, tumor aggressiveness and cancer mortality. I further highlight published studies that suggest a role for insulin as a potential mediator by which obesity and cancer could be linked.

### Effects of obesity on cancer risk and progression

Epidemiological studies have indicated that obesity is a risk factor for cancers of several tissues; obesity is associated with cancers of the esophagus, thyroid, colon, kidney, and liver in both men and women (Larsson and Wolk, 2007; Renehan et al., 2008); with rectal cancers in men (Renehan et al., 2008); and with endometrial, gallbladder, postmenopausal breast, and pancreatic cancers, and with brain and/or central nervous system tumors and gliomas in women (Renehan et al., 2008; Sergentanis et al., 2015). Obesity is a risk factor for diabetes (Abdullah et al., 2010), a chronic metabolic disease characterized by elevated levels of circulating glucose, and people with diabetes have a similarly increased risk of developing several types of cancers, including those of the colon (Larsson et al., 2005), breast (Larsson et al., 2007), pancreas (Huxley et al., 2005), liver (El-Serag et al., 2006) and endometrium (Friberg et al., 2007). The effect of metabolic disease on prostate cancer is controversial; studies have reported both tumor-promoting and -suppressing effects (Kasper and Giovannucci, 2006; Ma et al., 2008). Obesity also promotes tumor aggressiveness; obese individuals with progesterone-receptor-negative breast cancer have a higher risk of lymph node metastasis (Maehle et al., 2004). Furthermore, obesity leads to higher overall cancer-related mortality rates, and 15-20% of all cancer-related deaths in the United States are thought to be attributable to the affected individuals being overweight or obese (Calle et al., 2003). Similarly, in a large cohort study, diabetes has been demonstrated to be an independent predictor of mortality associated with colon, pancreatic and breast cancer, and, in men, cancer of the liver or bladder (Coughlin et al., 2004). A recent study indicates that pre-existing diabetes is associated with poor overall survival in women with lung cancer (Luo et al., 2016). Thus, a growing body of evidence suggests that obesity and its associated metabolic disease not only increases cancer risk but also accelerates malignant progression.

### Hyperinsulinemia: a mechanism underlying obesity-cancer interplay?

The hormonal effects of insulin are one potential mechanism by which obesity and cancer could be linked. Obesity is typically associated with organismal insulin resistance, a systemic condition whereby tissues fail to respond to insulin (Kahn and Flier, 2000). To compensate for insulin resistance, levels of insulin in the blood rise, leading to chronic hyperinsulinemia. Increased circulating insulin levels have been identified as a risk factor for the development of hepatocellular carcinoma (Donadon et al., 2009) and colorectal cancer (Kaaks et al., 2000; Yoon et al., 2015). Together with the well-documented mitogenic effects of insulin (Ish-Shalom et al., 1997), these studies lead to the hypothesis that increased insulin levels might play an important role in tumor formation and progression in obese individuals. However, the development of insulin resistance in obesity raises the question of how tumors utilize increased levels of circulating insulin in an insulin-resistant environment, a question I return to later in this article.

## *Drosophila* models of diet-induced obesity

**Fig. 1.**
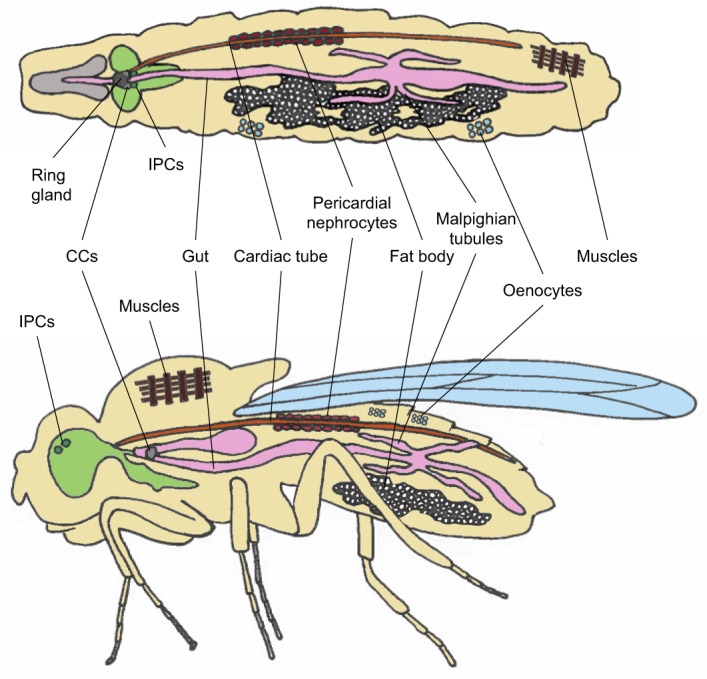
**Schematic of *Drosophila* tissues that contribute to metabolic homeostasis.** Schematic of (top) a dorsal view and (bottom) a lateral view of an adult *Drosophila*, showing the cells, tissues and organs involved in metabolism. Insulin-producing cells (IPCs) regulate carbohydrate homeostasis, similar to mammalian pancreatic β cells. Corpora cardiaca cells (CCs) – in the ring gland of the larvae and at the junction between the crop and the gut in adults – regulate carbohydrate and lipid homeostasis. The gut regulates nutrient digestion and absorption. The fat body serves as a primary glycogen and lipid storage organ, similar to adipose tissue and liver in mammals. Oenocytes accumulate mobilized lipids from the fat body upon starvation and have a similar function in this regard to mammalian hepatocytes. The cardiac tube promotes nutrient and hormone circulation. Pericardial nephrocytes regulate waste filtration, similar to mammalian kidney podocytes. Malpighian tubules regulate waste excretion and osmoregulation, similar to mammalian renal tubules.

*Drosophila* has become an increasingly popular model system in which to study metabolic homeostasis. Many of the metabolic enzymes and pathways that control glucose homeostasis are highly conserved between flies and higher organisms (Alfa and Kim, 2016). In *Drosophila*, circulating carbohydrate levels are regulated and maintained by two clusters of neurosecretory cells: insulin-producing cells (IPCs) and corpora cardiaca cells (CCs). IPCs in the brain are analogous to the vertebrate pancreatic β cells (Brogiolo et al., 2001; Broughton et al., 2005; Ikeya et al., 2002), whereas CCs located in the ring gland, a master endocrine organ, are analogous to the vertebrate pancreatic β cells (Lee and Park, 2004) (Fig. 1). IPCs and CCs secrete *Drosophila* insulin-like peptides and insect adipokinetic hormone – functional homologs of insulin and glucagon, respectively. The ablation of *Drosophila* CCs leads to hypoglycemia (Kim and Rulifson, 2004), whereas ablation of their IPCs leads to hyperglycemia (Broughton et al., 2005; Rulifson et al., 2002), confirming functional homology with their mammalian counterparts.

*Drosophila* and humans also have many of the other organs that control basic metabolic functions in common (Fig. 1). Nutrients are digested and absorbed in the gut, similar to the intestine in mammals (Lemaitre and Miguel-Aliaga, 2013). The fat body stores carbohydrates and lipids as glycogen and triacylglycerides (TAG), similar to adipose tissue and the liver in mammals (Arrese and Soulages, 2010). The fat body also functions as a nutrient sensor that remotely controls insulin production and secretion from the IPCs (Agrawal et al., 2016; Geminard et al., 2009; Rajan and Perrimon, 2012). Oenocytes are specialized secretory cells that function in a manner similar to the mammalian liver and that can mobilize lipid from the fat body upon starvation (Gutierrez et al., 2007).

Over the past decade, a series of studies has used flies to investigate metabolic homeostasis (reviewed in Owusu-Ansah and Perrimon, 2014). Genetic screens in flies have identified new regulators of metabolic homeostasis, including the sirtuin-family protein deacetylase Sir2 (Reis et al., 2010), the *Drosophila* Wnt protein Wingless (Lee et al., 2014), components of the Hedgehog signaling pathway (Pospisilik et al., 2010) and components of store-operated Ca^2+^ entry (Baumbach et al., 2014). More recently, flies have been increasingly used to model diet-induced obesity (Table 1). The two important dietary supplements used to induce obesity in fly models are fat and sugar, as I discuss in more detail below.

**Table 1.**
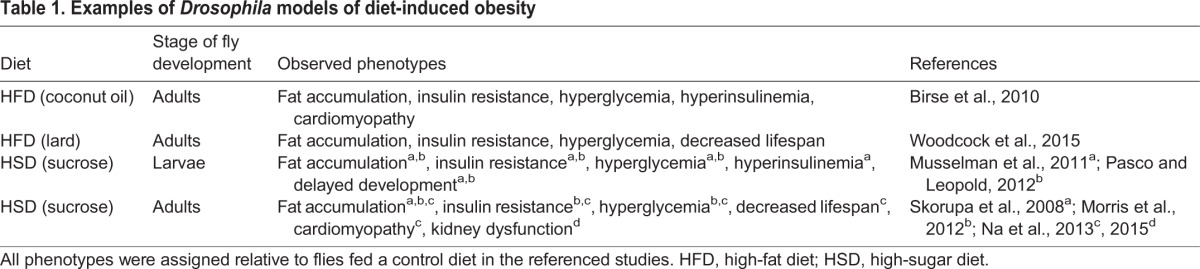
**Examples of *Drosophila* models of diet-induced obesity**

### High-fat diet model

The intake of a lipid-rich diet has been associated with human obesity (Bray and Popkin, 1998), and this is mirrored in flies. In one study, feeding adult flies a diet containing increasing amounts of fat (from coconut oil) led to excess fat accumulation in the adipose tissue (fat body) and in the midgut (Birse et al., 2010). In addition, these flies developed heart dysfunction (Birse et al., 2010; Diop et al., 2015), an important complication associated with obesity in humans. The insulin and target of rapamycin (TOR) pathways are highly conserved regulators that control growth and metabolism (Grewal, 2009). Systemic inhibition of the TOR pathway prevents high-fat diet (HFD)-induced obesity and cardiac dysfunction, indicating that deregulation of the insulin-TOR axis is responsible for deleterious HFD-induced effects (Birse et al., 2010). Another study used lard as a source of fat; feeding adult flies a lard-based HFD induced the accumulation of fat, a progressive increase in glucose levels (hyperglycemia), impaired insulin sensitivity (insulin resistance) and a decreased lifespan (Woodcock et al., 2015). A systemic activation of the Janus Kinase (JAK) and Signal Transducer and Activator of Transcription (STAT) signaling pathway, a downstream mediator of the cytokine signaling pathway (Li, 2008), was observed in the lard-based HFD-fed flies (Woodcock et al., 2015). It has recently been shown that the macrophage-specific inhibition of Unpaired 3 (Upd3), a cytokine that activates the JAK-STAT pathway, reverses insulin resistance in lard-fed flies and the associated reduction in their lifespan, indicating that macrophage-derived Upd3 is responsible for HFD-induced metabolic dysfunction (Woodcock et al., 2015).

### High-sugar diet model

High levels of dietary sugars have been linked to excess body weight in humans (Te Morenga et al., 2013). Expanding on a previous study that showed that excess dietary sugar increases fat accumulation in adult flies (Skorupa et al., 2008), *Drosophila* larvae fed a diet high in sucrose exhibited important aspects of obesity-related metabolic disorders, including fat accumulation, insulin resistance and hyperglycemia (Musselman et al., 2011). Increased circulating *Drosophila* insulin-like peptide (ILP)2 was also observed, as assessed by the use of FLAG-tagged ILP2 that had been overexpressed in the IPCs (Honegger et al., 2008; Musselman et al., 2011); a recently developed genetic tool to measure physiological levels of ILP2 in flies permits a more accurate and sensitive quantification of circulating ILP2 in diet-induced obesity models (Park et al., 2014). Other findings have demonstrated that fat storage in the fat body protects against the deleterious effects of a high-sugar diet (HSD); once the capacity of the fat body to store fat has been exceeded, the adverse metabolic consequences of this diet occur (Musselman et al., 2013). In a similar HSD-induced larval model, the *Drosophila* lipocalin-family member, neural lazarillo (NLaz) – which is analogous to apolipoprotein D and to retinol-binding protein 4 (Hull-Thompson et al., 2009), a protein implicated in diabetes in humans – has been identified as being a key mediator of HSD-mediated insulin resistance (Pasco and Leopold, 2012).

Adult flies fed a HSD also exhibit insulin resistance and the accumulation of fat (Morris et al., 2012; Na et al., 2013). These metabolic defects result in a reduced lifespan relative to that of flies raised on a control diet (Na et al., 2013). In addition, HSD-fed flies develop heart and kidney dysfunction, confirming that HSD-feeding reproduces important complications associated with human obesity (Na et al., 2013; Na et al., 2015). These findings highlight the potential utility of *Drosophila* diet-induced obesity models for the study of obesity-related disorders.

Altogether, flies challenged with high-calorie diets – based on fat or sugar – accumulate fat and develop metabolic defects similar to those observed in obese humans, including insulin resistance and hyperglycemia. These diet-induced *Drosophila* obesity models should help to inform our understanding of metabolic disease in humans. For example, a recent study used the HSD-induced obesity model in flies to evaluate candidate genes that had been identified previously in human genome-wide association studies (GWAS) of type 2 diabetes (Pendse et al., 2013). Although *Drosophila* cannot provide a perfect model of human metabolic diseases, it provides a useful system for exploring specific aspects of diet-induced metabolic dysfunction. Importantly, these diet-induced obesity models also allow us to study obesity-related disorders, including heart disease (Birse et al., 2010; Diop et al., 2015; Na et al., 2013), kidney disease (Na et al., 2015) and cancer (Hirabayashi et al., 2013; Hirabayashi and Cagan, 2015). Below, I discuss a fly model in which diet-induced obesity is combined with a model of cancer in order to study the biological link between obesity and cancer.

## A *Drosophila* model connecting obesity and cancer

*Drosophila* has also contributed to our understanding of the signaling pathways involved in tumor formation and progression (reviewed in Brumby and Richardson, 2005; Gonzalez, 2013; Hariharan and Bilder, 2006; Miles et al., 2011; Vidal and Cagan, 2006). Sophisticated genetic tools are available for use in *Drosophila* to perform genetic mosaic analysis using the FLP-FRT recombination system (see Box 1). This analysis allows researchers to investigate the interaction between mutant cells and wild-type cells within a tissue; the local cell-cell interactions are particularly important when studying aspects of cancer, including cancer migration, invasion and metastasis (reviewed in Miles et al., 2011; Ohsawa et al., 2014).

An important discovery made using the *Drosophila* genetic mosaic screen (see Box 1) was the identification of the Hippo pathway, a pathway that regulates tissue growth and cell fate (reviewed in Harvey and Tapon, 2007). The Hippo pathway regulates growth through the activation of Yorkie (Yki), a transcriptional co-activator that promotes proliferation and inhibits cell death (Huang et al., 2005). A large number of subsequent studies have demonstrated that the Hippo pathway is highly conserved in mammalian systems and that its deregulation occurs in various human cancers (reviewed in Pan, 2010; Yu et al., 2015).

*Drosophila* genetic mosaic screens have also helped to establish the concept of ‘cell competition’, a phenomenon initially observed in *Drosophila*, in which cells with different fitness levels compete for survival (Morata and Ripoll, 1975; Simpson and Morata, 1981; Igaki, 2015). *Drosophila* studies have subsequently discovered that the proto-oncogene Myc plays a key role in cell competition; cells that have higher Myc levels have a competitive growth advantage over neighboring cells with lower Myc levels (de la Cova et al., 2004, 2014; Johnston et al., 1999; Moreno and Basler, 2004). Because many oncogenes and tumor-suppressor genes are implicated in altering the competitive status of cells, cell competition is thought to play an important role in cancer (reviewed in Baker and Li, 2008; Moreno, 2008; Tamori and Deng, 2011; Wagstaff et al., 2013). These examples highlight important discoveries that have been made by using *Drosophila* genetic tools to identify new pathways and new concepts relevant to cancer. In the remainder of this section, I discuss a fly model that has been recently developed to study the interplay between obesity and cancer.

### A *Drosophila* model of Ras and Src co-activated tumors

Ras-family proteins control fundamental cellular processes, including cell growth, proliferation and survival. Ras-family proteins are frequently mutated and activated in various human cancers (Prior et al., 2012). Src tyrosine kinase also regulates cell proliferation, cell survival and cell migration. Src is activated in various human cancers (Irby and Yeatman, 2000). The combined elevation of Ras and Src activity is a common occurrence in several types of human cancer, including breast, colon and pancreatic cancers (Ishizawar and Parsons, 2004; Morton et al., 2010). More than 95% of individuals with pancreatic carcinoma harbor an activating mutation in *KRAS* (Almoguera et al., 1988). In individuals with pancreatic ductal adenocarcinoma (PDA), Src expression and activation have been found to be increased in 75% and 60% of tumors, respectively (Lutz et al., 1998; Morton et al., 2010). KRAS and Src co-activation in mouse pancreas causes PDA to develop with a shorter latency than in other tested oncogenic combinations, indicating that Ras and Src activation act synergistically (Shields et al., 2011).

To model Ras and Src co-activated tumors in flies, an oncogenic isoform of the fly Ras ortholog, Ras85D (Ras1), has been combined with a null mutant allele for *Drosophila* C-terminal Src kinase (Csk). Csk is a negative regulator of Src; its loss therefore leads to the activation of Src (Pedraza et al., 2004; Read et al., 2004). The mosaic analysis with a repressible cell marker (MARCM) technique (Lee and Luo, 1999) (see Box 1) was used to create clones of cells within the developing eye epithelial tissue that are homozygous mutant for *Csk* and that simultaneously express an oncogenic form of *ras1*, *ras1^G12V^* (thus with the genotype, *ras1^G12V^;csk^−/−^*). These Ras and Src co-activated cells were labeled with GFP to visualize tumor progression *in situ* (Hirabayashi et al., 2013).

**Fig. 2.**
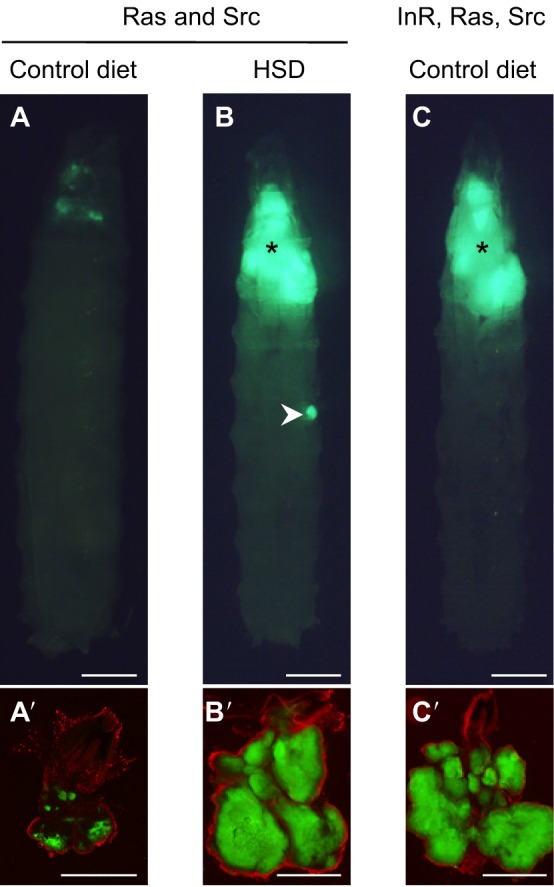
**The use of *Drosophila* to study the obesity–cancer connection.** (A-C) A dorsal view of a third-instar *Drosophila* larva. Anterior is at the top, posterior at the bottom. Scale bars: 500 μm. (A) A *ras1^G12V^;csk^−/−^* (Ras and Src) larva raised on a control diet. Ras and Src co-activated cells (labeled with GFP; green) develop benign tumors within the eye epithelial tissue. (B) A *ras1^G12V^;csk^−/−^* (Ras and Src) larva raised on a HSD. Ras and Src co-activated cells (labeled with GFP; green) develop a large primary tumor (asterisk) associated with secondary tumors (arrowhead). (C) An *inr^CA^, ras1^G12V^;csk^−/−^* (InR, Ras, Src) larva raised on a control diet. InR, Ras and Src co-activated cells (labeled with GFP; green) develop into a large primary tumor (asterisk) but not into secondary tumors. (A′-C′) Dissected eye epithelial tissue from each of the corresponding fly mutants (A-C) stained with DAPI (red) to outline the tissue. Scale bars: 500 μm. Images are modified from Hirabayashi and Cagan, 2015, licensed under a Creative Commons Attribution 4.0 International license. *csk*, C-terminal Src kinase gene; GFP, green fluorescent protein; InR, insulin receptor; *inr^CA^*, constitutively active allele of the insulin receptor gene; *ras1^G12V^*, oncogenic allele of *ras1*.

In *ras1^G12V^;csk^−/−^* animals fed a control diet, Ras and Src co-activated cells generate small tumors within the eye epithelial tissue (Fig. 2A). Previous studies have demonstrated that Src-activated cells undergo apoptotic cell death when surrounded by wild-type cells (Enomoto and Igaki, 2013; Vidal et al., 2006). The activation of Ras in this model, however, cannot completely overcome Src-mediated cell death, and so only a proportion of the Ras and Src co-activated cells that are adjacent to wild-type cells undergo apoptotic cell death. As a result, Ras and Src co-activated cells do not overgrow in this model, but instead develop into multiple benign tumors (Fig. 2A).

### Combining *Drosophila* models of diet-induced obesity and cancer

A fly model of HSD-induced obesity has been used to explore the effects of obesity on Ras and Src co-activated tumor progression *in situ* (Hirabayashi et al., 2013). When *ras1^G12V^;csk^−/−^* flies are fed a HSD, the Ras and Src co-activated cells resist apoptotic cell death and develop into large tumors that are associated with emergent metastases and with secondary tumors (Fig. 2B). This diet-enhanced Ras and Src co-activated tumor model has also been used to investigate whether increased insulin levels underlie the obesity-mediated promotion of malignant tumors.

**Fig. 3.**
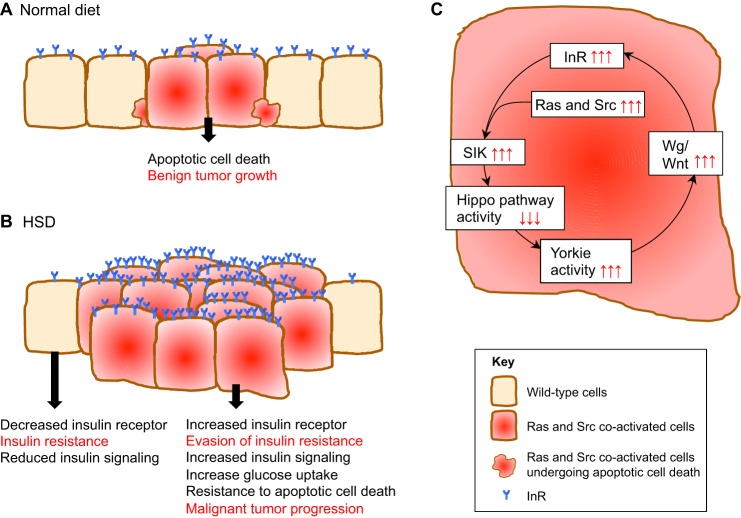
**Summary of interactions between diet-induced obesity and tumor growth in *Drosophila*.** (A) Under conditions of a normal diet, Ras and Src co-activated cells develop into benign localized tumors. A proportion of Ras and Src co-activated cells that are adjacent to the wild-type cells undergo apoptotic cell death. (B) Under conditions of a HSD, wild-type cells develop insulin resistance, whereas Ras and Src co-activated cells evade insulin resistance, promote increased insulin signaling and resist apoptotic cell death, leading to malignant tumor progression. (C) Ras and Src co-activated tumors promote malignant tumor progression through a SIK-Yki-Wg-InR signaling circuit. Diet-induced obesity promotes SIK activity in Ras and Src co-activated cells, leading to the downregulation of the Hippo signaling pathway. This results in increased Yki activity and in increased Wg expression. Increased Wg signaling increases expression of the InR, leading to increased insulin sensitivity and to the evasion of insulin resistance. Increased insulin signaling in Ras and Src co-activated cells further promotes SIK activity, forming a feed-forward circuit. InR, insulin receptor; SIK, salt-inducible kinase; Wg, Wingless; Yki, Yorkie.

When wild-type flies are fed a HSD, it causes wild-type eye tissue to develop insulin resistance that is associated with the reduced expression of the insulin receptor (InR) (Hirabayashi et al., 2013). However, in *ras1^G12V^;csk^−/−^* flies, Ras and Src co-activated tumors in the eye tissue not only retain insulin sensitivity but become hyper-reactive to insulin, leading to increased insulin–PI3K signaling and to enhanced glucose uptake (Hirabayashi et al., 2013). A HSD increases canonical Wingless (Wg)/Wnt signaling, specifically in Ras and Src co-activated tumors, which in turn increases InR expression, resulting in the evasion of diet-mediated insulin resistance. In addition, the expression of a constitutively active isoform of InR in Ras and Src cells (*inr^CA^,ras1^G12V^;csk^−/−^*) (Fig. 2C) is sufficient to elevate Wg/Wnt signaling and to promote tumor overgrowth, even with a control diet. These results have revealed the existence of a signaling circuit with a ‘feed-forward’ mechanism, whereby Ras and Src co-activation, and increased insulin-PI3K signaling promote elevated InR expression through Wg/Wnt signaling. Through this circuit, tumors that evade insulin resistance in obese flies exhibit strongly enhanced glucose uptake and progression to malignant tumors (Fig. 3A,B).

These findings highlight that a connection exists between canonical Wg/Wnt signaling and insulin signaling. A functional link between canonical Wg/Wnt and insulin signaling pathways is also conserved in humans. A study involving human cells has demonstrated that the Wnt co-receptor, LDL receptor-related protein 6 (LRP6), promotes InR expression through canonical Wnt signaling (Singh et al., 2013a,b). In addition, a study using human pre-adipocytes has revealed that cross-talk exists between the insulin signaling and Wnt signaling pathways at multiple levels (Palsgaard et al., 2012).

### Salt-inducible kinase mediates tumor growth in obese animals

In the Ras and Src co-activated tumors of diet-induced obese *Drosophila*, elevated Wg signaling is a key mediator that promotes the evasion of insulin resistance and tumor progression. Salt-inducible kinase (SIK; of which there are two family members in *Drosophila*) has recently been identified as an upstream mediator of increased Wg expression in diet-induced Ras and Src co-activated tumors (Hirabayashi and Cagan, 2015). SIK is a serine/threonine kinase of the AMP-kinase family that regulates metabolic homeostasis in both fly (Choi et al., 2011, 2015; Wang et al., 2008, 2011) and mammalian systems (Dentin et al., 2007; Patel et al., 2014). More recently, SIK has been identified as a negative regulator of Hippo signaling in *Drosophila*; it inhibits the Hippo pathway, leading to the activation of the transcriptional co-activator Yki (Wehr et al., 2013). Yki promotes the expression of genes that regulate cell proliferation and differentiation, including Wg (Cho et al., 2006). In Ras and Src co-activated tumors, increased insulin signaling promotes SIK activity, revealing the SIK-Yki-Wg axis as the feed-forward circuit that reverses insulin resistance (Fig. 3C) (Hirabayashi and Cagan, 2015). These results indicate that SIK acts as a nutrient sensor that links insulin availability to Yki-mediated evasion of insulin resistance and tumor growth. Through this mechanism, Ras and Src co-activated cells undergo tumorigenic growth in nutrient-rich conditions, such as in obesity. Taken together, the combined *Drosophila* Ras and Src co-activated tumor model, as well as the feeding-based obesity model, have provided new mechanistic insights into how tumors tune their metabolism to take advantage of increased insulin and glucose levels, and to thrive in conditions of organismal insulin resistance.

### Model limitations

As emphasized throughout this Review, *Drosophila* models have various advantages over other models. However, there are limitations to these models. Flies are not small humans, and follow-up studies in mammalian systems are ultimately essential for validating findings from fly studies. Reduced genetic redundancy in flies allows the functions of disease-causing genes to be characterized with greater efficiency. However, this also represents a limitation as the fly might lack specific gene-regulatory mechanisms that are present in mammalian systems. For example, the insulin and insulin-like growth factor-1 (IGF-1) signaling pathway is more complex in higher organisms (Poloz and Stambolic, 2015), and findings from fly studies thus require careful assessment for their applicability to such organisms. Despite these limitations, *Drosophila* has already proven to be a useful model system for identifying therapeutic compounds. In the next section, I highlight some examples of chemical screening performed in *Drosophila* models of human disease and discuss how this approach has been used to identify potential targets that link obesity and cancer.

## Breaking the connections between obesity and cancer

One of the long-term goals of disease studies is to advance the development of new therapeutics. One considerable advantage of using flies to model disease is the ability to test compounds in a whole-animal setting. Whole-animal compound screening can both identify drugs with multiple targets and also eliminate drugs that produce significant *in vivo* toxicity. In addition, whole-animal screening can identify distinct classes of drugs that would not otherwise be possible to identify using cancer cell lines *in vitro*, such as drugs that act both on the tumor and elsewhere in the body. Fly models in particular provide a rapid and inexpensive means of accelerating drug discovery in this way.

### Chemical screening in *Drosophila*

A robotics-based approach, combined with raising flies in a 96-well format, is increasingly being used to screen large numbers of compounds in *Drosophila* models of human disease, including models of Fragile X syndrome (Chang et al., 2008), myotonic dystrophy (Garcia-Alcover et al., 2014) and cancer (Levine and Cagan, 2016; Willoughby et al., 2013). A compound identified through chemical screening in a fly model of cancer is already in clinical use. ZD6474 (vandetanib) has been identified as a candidate compound that suppresses tumor growth in a fly model of multiple endocrine neoplasia type 2 (MEN2) (Read et al., 2005; Vidal et al., 2005). Based primarily on tissue-culture-derived (Carlomagno et al., 2002) and fly data, ZD6474 (vandetanib) entered clinical trials and was later approved as the first medication for metastatic medullary thyroid cancer (Thornton et al., 2012). This example provides a proof-of-principle that a whole-animal *Drosophila* approach could be used to identify useful cancer therapeutics. Furthermore, as recently demonstrated using the *Drosophila* MEN2 model, fly genetics have been used to identify the optimal inhibition profile of kinase inhibitors, creating a unique opportunity for rational drug development by improving efficacy and reducing whole-animal toxicity (Dar et al., 2012).

### Using a diet-enhanced Ras and Src co-activated tumor model to identify potential therapeutics

Most *ras1^G12V^;csk^−/−^* animals fed a HSD die as larvae owing to enhanced tumorigenesis (Hirabayashi et al., 2013). Survival to the pupal stage (i.e. rescue from larval lethality) is useful for making a quantitative assessment of the efficacy of a compound with respect to tumor suppression, as well as for monitoring whole-animal toxicity. A rational chemical screen using this model has identified a cocktail of compounds that target: (i) dietary sucrose (acarbose); (ii) the Wg/Wnt signaling pathway (pyrvinium) (Thorne et al., 2010); and (iii) the Ras-Src-insulin signaling pathway (AD81, a multi-kinase inhibitor targeting Raf, Src and S6-kinase) (Dar et al., 2012). Feeding *ras1^G12V^;csk^−/−^* flies a HSD that contained each of these compounds separately mildly suppresses tumor growth. By contrast, feeding them all three compounds together dramatically suppresses tumor growth, allowing most flies to develop to the pupal stage (Hirabayashi et al., 2013). These results demonstrate that rational combinatorial therapy can provide optimal efficacy with minimal whole-animal toxicity.

## Outstanding questions

*Drosophila* studies have provided mechanistic insights into the roles of insulin in cancer risk and progression linked to obesity. However, many questions have emerged from these studies. Here, I highlight some of the key outstanding questions.

### The quality of diet, obesity and cancer

The quality of fat has an important effect on the development of insulin resistance. A large-scale human intervention study has reported that saturated fat significantly impairs insulin resistance, which remain unchanged on a monounsaturated-fat diet (Vessby et al., 2001). Notably, a HFD (calorie-matched to a HSD) only promotes a moderate level of insulin resistance in the larvae-feeding model, indicating that the HFD does not promote metabolic dysfunction (Musselman et al., 2011). Consistent with this, the HFD fails to promote tumorigenesis in the Ras and Src co-activated tumor model (Hirabayashi et al., 2013). Both studies used vegetable shortening (Crisco; 25% saturated fat) as a source of fat (Hirabayashi et al., 2013; Musselman et al., 2011). Other studies have induced obesity in the adult fly by using fat with higher saturation profiles: coconut oil (86% saturated fat) (Birse et al., 2010) or pork lard (44% saturated fat) (Woodcock et al., 2015). Whether the difference in the quality of dietary fat results in different cancer outcomes remains an important question.

One of the crucial issues with respect to exploring dietary effects in animals is inconsistency in the base diets used in the studies. The recent development of a chemically defined diet for *Drosophila* would thus be an invaluable base medium for better-defined experiments (Piper et al., 2014; Reis, 2016). The future use of chemically defined diets could thus provide more definitive answers to questions regarding the relationships between diet quality, obesity and cancer.

### Relationship between obesity and cancer mutations

Although epidemiological studies have revealed specific cancers to be associated with obesity, little is known about the oncogenic characteristics of the tumors that are linked to obesity. An important question to have emerged from fly studies is whether the ability of tumors to evade diet-induced insulin resistance is specific to Ras and Src co-activated tumors. A recent study has found that obesity promotes the growth of ETS-related gene (*ERG*)-overexpressing prostate tumors but not that of *ERG*-negative tumors (Pettersson et al., 2013). This implies that tumors harboring different oncogenic signatures might have different levels of sensitivity to obesity.

In contrast to the tumor-promoting effects induced by high-calorie diets, dietary restriction (DR) decreases the growth of various types of tumors in rodent models through reduced systemic insulin and IGF-1 signaling (Breese et al., 1991; Ruggeri et al., 1989). A recent study has indicated that tumors with PI3K activation are resistant to the tumor-suppressing effects of DR (Kalaany and Sabatini, 2009). Similarly, differential PI3K activity in tumors could contribute to their different levels of sensitivity to obesity – i.e. tumors harboring PI3K-pathway-activating mutations might be resistant to the tumor-promoting effects of obesity, whereas tumors without PI3K pathway activation might benefit from the tumor-promoting effects of hyperinsulinemia. Sequencing the tumors that arise in obese individuals would therefore provide important information on whether obesity promotes cancers with specific mutational profiles.

### Is cell competition involved in the obesity-cancer connection?

Diet-enhanced Ras and Src co-activated tumors promote Yki activation, leading to the increased expression of Wg and Myc (Hirabayashi and Cagan, 2015). Increased Myc (de la Cova et al., 2004; Johnston et al., 1999; Moreno and Basler, 2004), Wg (Vincent et al., 2011) and Yki (Suijkerbuijk et al., 2016) levels can turn cells into ‘super-competitors’, inducing apoptotic cell death of the surrounding wild-type cells and allowing the super-competitor cells to expand and colonize the tissue. Obesity-enhanced tumorigenesis initiated by co-activation of Ras and Src could therefore potentially be explained by cell competition. Whether obesity-enhanced Ras and Src co-activated tumors actively induce apoptotic cell death and eliminate the surrounding wild-type cells has not been clearly demonstrated. Additional studies will be required to confirm whether obesity-enhanced Ras and Src co-activated tumorigenesis involves cell competition.

### Mechanism of obesity-enhanced metastasis

In the *ras1^G12V^;csk^−/−^ Drosophila* model, metastases and secondary tumor formation are observed in flies raised on a HSD (Fig. 2B) (Hirabayashi et al., 2013). The *inr^CA^,ras1^G12V^;csk^−/−^* flies fed a control diet develop tumors to a similar extent as that seen in *ras1^G12V^;csk^−/−^* flies fed a HSD, but these tumors fail to undergo metastases or secondary tumor formation (Fig. 2C) (Hirabayashi et al., 2013). This suggests that increased insulin signaling within the tumor is insufficient to promote metastasis. Increased levels of circulating insulin and/or glucose might elicit additional effects on tumor migration and metastasis. A recent study has demonstrated that high glucose levels enhance the ability of tumor cells to migrate and to metastasize (Chocarro-Calvo et al., 2013). Further investigation into this issue will be important for understanding the effects of obesity on the progression of malignant tumors.

### Is obesity-associated cancer reversible?

Obesity can be reversed by dietary modification and by increased exercise. However, whether obesity-induced cancer can be reversed is unknown. An associated intriguing question is whether tumors that have responded to obesity become addicted to sugar. Would switching back to a control diet after the tumors have become enhanced by obesity be sufficient to cause their regression? Is there a point of no return? Exploring these issues could have therapeutic implications for the development of treatment strategies for patients with obesity-associated cancers.

## Conclusions

This Review highlights the use of *Drosophila* as a model organism for studying the connections between obesity and cancer. *Drosophila* studies have provided conceptual and mechanistic advances in our understanding of the role that insulin plays in obesity-related cancers. As I have discussed, many unanswered questions remain. Multiple diet-induced obesity models (Table 1) and an increasing number of tissue- and organ-specific cancer models are available in *Drosophila* (Table 2). Combining these different models should enhance our insight into the biological mechanisms that connect diet, obesity, insulin resistance and cancer. In addition, *Drosophila* has proven to be a useful whole-animal platform for drug discovery. I anticipate that *Drosophila melanogaster* will continue to be an important model system for investigating the obesity-cancer connection and will likely yield new directions for future research.

**Table 2.**
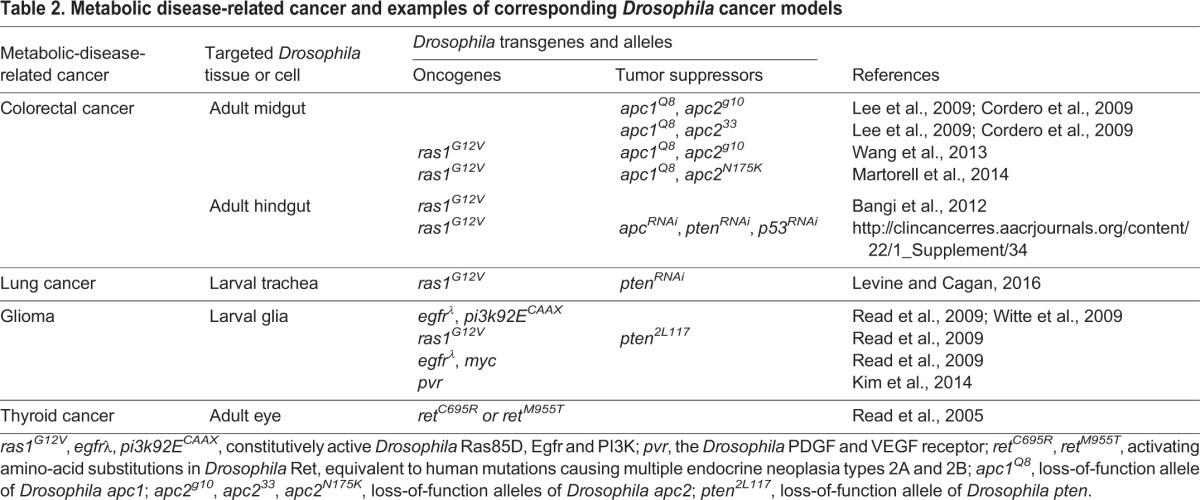
**Metabolic disease-related cancer and examples of corresponding *Drosophila* cancer models**
